# Systemic inflammation is associated with differential neural reactivity and connectivity to affective images

**DOI:** 10.1093/scan/nsaa065

**Published:** 2020-05-14

**Authors:** Gabriella M Alvarez, Daniel A Hackman, Adam Bryant Miller, Keely A Muscatell

**Affiliations:** Department of Psychology & Neuroscience, University of North Carolina at Chapel Hill, Chapel Hill, NC 27599-3270, USA; USC Suzanne Dworak-Peck School of Social Work, University of Southern California, Los Angeles, CA 90089, USA; Department of Psychology & Neuroscience, University of North Carolina at Chapel Hill, Chapel Hill, NC 27599-3270, USA; Department of Psychology & Neuroscience, University of North Carolina at Chapel Hill, Chapel Hill, NC 27599-3270, USA; Lineberger Comprehensive Cancer Center, University of North Carolina at Chapel Hill, Chapel Hill, NC 27514, USA

**Keywords:** inflammation, affect, functional connectivity, hippocampus, amygdala, interleukin-6

## Abstract

Systemic inflammation is increasingly appreciated as a predictor of health and well-being. Further, inflammation has been shown to influence and be influenced by affective experiences. Although prior work has substantiated associations between inflammatory and affective processes, fewer studies have investigated the neurobiological correlates that underlie links between systemic, low-grade inflammation and affective reactivity. Thus, the current study examined whether markers of systemic inflammation (i.e. interleukin-6, C-reactive protein) are associated with differential patterns of neural activation and connectivity in corticolimbic regions in response to affective images. We investigated this question in a sample of 66 adults (44 women, M age = 54.98 years, range = 35–76) from the Midlife in the United States study. Higher levels of inflammation were associated with lower activity in limbic regions (i.e. amygdala, hippocampus, anterior insula, temporal pole) when viewing positive (*vs* neutral) images. Higher levels of inflammation were also associated with greater connectivity between the hippocampus and the medial prefrontal cortex in response to positive images. Inflammatory markers were not associated with significant differences in activation or connectivity to negative images. These findings highlight the utility of health neuroscience approaches in demonstrating that physiological processes such as inflammation are related to how our brains respond to affective information.

Systemic inflammation, a component of the innate immune system, is increasingly appreciated for its role in the pathophysiology of chronic disease and psychopathology (Dantzer *et al*., 2008; Liu *et al.,* 2017; Bennett *et al.,* 2018). The inflammatory response is primarily an adaptive defense mechanism that activates to harmful pathogens and promotes healing; however, chronic and uncontrolled inflammation can have negative consequences for physical and mental health (Gabay, 2006; Franceschi and Campisi, 2014; Furman *et al.,* 2019). Interestingly, a growing literature in psychoneuroimmunology shows that, in addition to its role in both acute infection and chronic disease, systemic inflammation both affects and is affected by psychological experiences (Dickerson *et al.,* 2009; Irwin and Cole, 2011). The purpose of the present study was to investigate the association between systemic, low-grade inflammation and one such psychological experience, affective reactivity. Specifically, we examined the relationship between markers of inflammation and neural responses to positive and negative images, compared to neutral images, to further our nascent understanding of the bidirectional links between the innate immune system and brain function.

What do we currently know about links between affective reactivity and inflammation? Most prior work in this area has focused on the links between negative affect, both chronic (e.g. depression) and acute (e.g. in response to a psychological stressor), and inflammation. Meta-analytic evidence suggests that elevated depressive symptoms are associated with higher levels of systemic inflammation (Howren *et al.,* 2009) and that acute stressors elicit increases in markers of systemic inflammation (Marsland *et al.,* 2017b). A handful of functional MRI (fMRI) studies have investigated the neural correlates of negative affect-inducing experiences and inflammation, showing that both social evaluation (Muscatell *et al*., 2016b) and grief elicitation among recently bereaved individuals (O’Connor *et al*., 2009b) are associated with greater activation in the medial prefrontal cortex, amygdala and anterior cingulate cortex and with greater levels of inflammation.

Some work has also investigated the ‘bottom-up’, afferent influence of inflammation on negative affective processes. This area of work demonstrates that experimentally induced increases in markers of inflammation (via inflammatory challenge studies utilizing lipopolysaccharide or typhoid vaccination) are associated with higher depressive symptoms and greater feelings of social disconnection (Eisenberger *et al.,* 2009, 2010b; Harrison *et al.,* 2009). Complementary neuroimaging work has investigated the influence of peripheral inflammation on neural reactivity to negative affect-related stimuli, such as threatening faces (Inagaki *et al.,* 2012), negative social feedback (Muscatell *et al*., 2016b), social exclusion (Eisenberger *et al.,* 2009) and threatening images (Kullmann *et al.,* 2013). Together, these studies show that higher levels of inflammation are associated with increased activity in limbic (e.g. amygdala, hippocampus) and cortical (e.g. medial prefrontal cortex, cingulate cortex) regions in response to negative stimuli. Thus, a growing literature shows that systemic inflammation both affects and is affected by negative emotional experiences and stimuli, via alterations in subcortical (i.e. amygdala) and cortical (i.e. ACC, insula, dmPFC) neural activity.

Fewer studies have explored the bidirectional links between positive affect and levels of inflammation, especially as they relate to neural functioning. This paucity of work is surprising, considering that several studies have found that both dispositional positive affect (Marsland *et al.,* 2006; Stellar *et al.,* 2015; Hartanto *et al.,* 2019) and momentary positive affective states (Steptoe *et al.,* 2005) are associated with lower levels of inflammation. To date, no known neuroimaging studies have examined the efferent pathway or how the induction of a positive affective experience influences levels of inflammation. Correlational studies show that activity in the medial prefrontal cortex in response to positive stimuli (e.g. favorite actor; positive autobiographical memories) is related to better innate immune system functioning (i.e. natural killer cell count; Matsunaga *et al.,* 2008) and lower inflammation (i.e. interferon-γ; Matsunaga *et al.,* 2013), respectively, suggesting that greater neural responses to positive stimuli might be related to lower levels of systemic inflammation. A more substantial literature has examined how manipulating inflammation results in changes in neural responses to positive affective stimuli. Most of the work examining this afferent pathway has focused on documenting inflammation-related changes in neural reactivity to monetary reward tasks. These studies generally find that inflammation causes a decrease in neural activity in reward-related regions (i.e. ventral striatum) in response to monetary gain (Eisenberger *et al*., 2010a; Capuron *et al.,* 2012; Moieni *et al.,* 2019, c.f. Inagaki *et al.,* 2015; Muscatell *et al*., 2016b). Together, these studies suggest that higher levels of inflammation might be associated with decreased activity in regions within the basal ganglia in response to positive stimuli. Generally, a growing literature demonstrates that systemic inflammation can both affect and be affected by positive experiences and stimuli, via alterations in subcortical (i.e. ventral striatum) and cortical (i.e. medial prefrontal cortex) neural activity, although less work has been conducted in this area.

Interestingly, there is substantial overlap in the brain regions that are implicated in inflammatory processes reviewed previously and in regions that show significant activation to positive and negative stimuli. For example, recent meta-analytic work has revealed that activity in several corticolimbic regions [e.g. dorsal medial prefrontal cortex (dmPFC), amygdala, hippocampus, striatum, insula] is consistently associated with levels of peripheral inflammation (Kraynak *et al.,* 2018). In another meta-analysis that examined the brain basis of affective processing (Lindquist *et al.,* 2016), similar limbic (e.g. amygdala, insula, striatum) and cortical regions (e.g. dmPFC and dACC) were also implicated in the processing of positive and negative information. Findings from these two meta-analyses converge to suggest that corticolimbic regions are involved in both affective and inflammatory processes. Thus, these corticolimbic regions may be important in facilitating cross-talk between the brain and the innate immune system in response to affective information.

Although prior research has identified associations between peripheral inflammation and corticolimbic activity in response to affective experiences, numerous gaps in our knowledge still exist. For example, most studies have utilized acute inflammatory challenge manipulations to study links between inflammation and neural activity; as such, we have limited knowledge about the association between chronic, low-grade inflammation and corticolimbic activity to affective stimuli. Further, most studies focus on monetary rewards as a proxy for positive experiences and angry/fearful faces for negative stimuli, leaving gaps in our knowledge of the associations between inflammation and neural responses to other types of affective stimuli (e.g. positive and negative scenes). Finally, although several psychoneuroimmunological studies have examined the associations between inflammation and functional connectivity while individuals are at rest (Felger *et al.,* 2016; Lekander *et al.,* 2016; Marsland *et al*., 2017a; Mehta *et al.,* 2018; Kraynak *et al.,* 2019; Nusslock *et al.,* 2019), few known studies have examined how markers of systemic inflammation might relate to functional connectivity while participants are engaged in a dynamic affective reactivity task. In several studies, task-based connectivity has been shown to outperform resting-state models for detecting relationships between neural activity and individuals’ differences in behavior (Greene *et al.,* 2018; Jiang *et al.,* 2020), suggesting that investigations of associations between inflammation and task-based connectivity are warranted. Thus, the present study was designed to fill these gaps in our knowledge by exploring associations between low-grade peripheral inflammation and neural reactivity and connectivity in response to viewing affective images.

To accomplish this, we examined associations between markers of systemic inflammation and neural reactivity/connectivity to affective images in a sample of 66 adults from the Midlife in the United States (MIDUS) study. Specifically, we examined the relationship between levels of interleukin-6 (IL-6) and C-reactive protein (CRP) and corticolimbic responsivity and connectivity to positive and negative images. IL-6 and CRP are two commonly measured markers of inflammation in psychoneuroimmunology research. IL-6 is an inflammatory cytokine that is released into circulation in response to both physical and psychological threats to help facilitate communication among immune cells, among other functions. IL-6 also stimulates the production of CRP, an acute-phase protein produced by the liver that plays several roles during an inflammatory response. Elevated levels of IL-6 and CRP in the absence of acute infection are often conceptualized as representing chronic, low-grade inflammation (O’Connor *et al*., 2009a).

## Methods

### Participants

Data for this project were drawn from the Midlife in the United States (MIDUS) study, a national longitudinal study that examines biopsychosocial factors influencing health across later life. A subset of individuals from the MIDUS cohort completed the Neuroscience Project, beginning in 2007. Participants were eligible for this sub-study if they completed the Biomarker Project 4 visit, met MRI inclusion criteria (e.g. no metal implants, no claustrophobia, not currently pregnant) and had no prior history of a neurological disorder. Of the 72 total individuals enrolled in the Neuroscience Project, for the present paper, we excluded six: two for excessive head motion, one due to incomplete fMRI data and four due to levels of CRP greater than 10 mg/l which likely indicates a current or recent infection (Jaye and Waites, 1997). The 66 participants included in the analyses had a mean age of 54.98 years (s.d. = 10.76; range = 35–76) and consisted of 44 women (66.67%). See Table 1 for additional demographic information. Participants in the fMRI subsample were of similar age and exhibited comparable values of CRP and IL-6 to those in the larger MIDUS study, such that there were no significant differences between the samples for these characteristics (*P*’s > 0.42).

**Table 1:** Demographic and biomarker summary of the study sample

Variable	Count (*N*)	Percentage (%)
Sex (female, %)	44	66.7
Race/ethnicity		
Black	19	28.8
Native American or Aleutian Islander	1	3
White	45	68.2
Other	1	3
Educational attainment		
Less than high school	27	40.9
High school	21	31.8
Bachelor’s degree	8	12.1
Master’s degree	10	15.2
	Mean (SD)	Range
Age	54.98 (10.76)	35–76
BMI	29.52 (5.67)	19.51–46.78
IL-6 (pg/ml)	2.88 (2.87)	0.16–18.40
IL-6 (natural log)	0.74 (0.81)	−1.83–2.91
CRP (ug/ml)	2.21 (1.98)	0.16–7.62
CRP (natural log)	0.38 (0.96)	−1.83–2.03

### Procedures and materials

#### Overview

Participants in this study first completed the Biomarker Project 4 visit. The visit was an overnight session in which participants completed questionnaires and provided urine, blood and saliva samples to assess biological indicators of physiological functioning and health status, including markers of inflammation. Following the biomarker collection, participants then completed an fMRI scan.

#### IL-6 and CRP

After overnight fasting, participants provided blood samples later assayed for levels of IL-6 and CRP. Assays were conducted using commercially available kits according to manufacturer instructions. Both CRP and IL-6 assays showed acceptable inter-assay CVs (2.1–12.3%). More details are provided in Supplemental Materials. Both IL-6 and CRP values were natural log-transformed to adjust for the positive skew in the data. Finally, given the significant correlation between IL-6 and CRP in the present sample (*r* = 0.63, *P* < 0.001) and the known physiological association (i.e. IL-6 can stimulate the production of CRP), the natural log values of IL-6 and CRP were standardized and averaged to create a composite inflammation score to assess the combined associations between these inflammatory markers and neural activity. See Supplementary Materials for exploratory analyses separated by inflammatory markers.

#### fMRI task

For the fMRI task (data from which are also published in Heller *et al*., 2013; van Reekum *et al*., 2018), participants viewed 60 positive, 60 negative and 60 neutral pseudo-randomized images selected from the International Affective Picture System (IAPS; Lang *et al*., 2008) for five runs (see Supplementary Materials for list of IAPS images used). The stimuli were matched across valence categories for complexity, social content, arousal and luminosity. Each trial progressed as follows: a fixation crosshair was displayed for 1 s, followed by an IAPS image presented for 4 s, and then a blank screen intertrial interval (ITI) was displayed (M length = 8.89 s, range = 5.5–17.6 s). Participants were instructed to indicate on a button box the valence (i.e. positive, negative, or neutral) of the image presented. After 40 out of 60 trials in each valence category, a neutral male face was presented for 0.5 s after the IAPS image was displayed. Because the current project focused on examining neural responses to the affective images, the face stimuli were coded as regressors of no-interest in analyses. Across runs, the order the valence of the images presented was consistent across participants, though the specific stimuli presented within each valence category were randomized across participants.

#### fMRI data collection

Neuroimaging data for the current study were collected on a GE SIGNA 3.0 Tesla high-speed MRI scanner with a standard clinical whole-head transmit–receive quadrature head coil. The blood oxygen level-dependent (BOLD) signal was acquired using a T2*-weighted gradient-echo echo-planar imaging (EPI) pulse sequence across five runs of approximately 8 min each. Each EPI acquired 30 sagittal slices that used the following parameters: TR = 2000 ms, TE = 30 ms, flip angle = 60°, field of view = 240 mm, acquisition matrix = 64 × 64 and 4 mm slice thickness with 1 mm gap. A T1-weighted anatomical image was also collected using a T1-weighted inversion recovery fast gradient echo with the following parameters: acquisition matrix = 256 × 256 and a field of view = 240 mm, with 124 × 1.1 mm axial slices.

### Data analysis

#### fMRI preprocessing and analysis

Neuroimaging data were preprocessed utilizing an in-house pipeline. The *fsl_motion_outliers* program (Jenkinson *et al.*, 2012) was used to identify artifacts and excessive motion. Motion spikes were included in each person-level general linear model (GLM) to control for motion exceeding 2 mm. Further, runs with 2 mm of framewise displacement for greater than 20% of volumes acquired were excluded (*N* = 10; 0.03% of total runs). Next, a four-dimensional registration algorithm utilizing NiPy to conduct spatio-temporal transformations that simultaneously motion and slice-time corrected (Roche, 2011) was implemented. In two steps, this algorithm aligned all five functional images to a mean image computed after initial realignment. FSL’s FLIRT algorithm coregistered T2*-weighted images to the T1-weighted images, which were then anatomically coregistered to each individual’s high-resolution structural image. Images were nonlinearly registered to the Montreal Neurologic Institute’s (MNI) standard space utilizing the Advanced Normalization Tools (ANTs) software (Avants *et al*., 2011). Finally, spatial smoothing was applied with a Gaussian kernel of 5-mm full width at half maximum.

fMRI data were analyzed using FSL’s FMRI Expert Analysis Tool (FEAT) Version 6.00. A general linear model (GLM) was constructed for each run per individual. The GLMs included regressors modeling the positive, negative and neutral events as well as the nuisance regressors of motion (i.e. each individual’s six motion parameters and their first derivatives and single-point motion outliers) and the face events. For each run, a high-pass filter (100 Hz) was applied to remove low-frequency drifts. Higher-level analyses were conducted, utilizing FLAME stage 1 (Woolrich *et al.*, 2004), a fixed-effects GLM approach, to combine BOLD activation and differences in variance across runs. The two contrasts of interests were negative images *vs* neutral images and positive images *vs* neutral images. The whole-brain main effects for both contrasts (cluster-based threshold at *z* > 2.3, *P* < 0.05) are reported in the Results section.

#### Region-of-interest construction

A mask was constructed by combining meta-analytic maps of neural activation related to affective processing (Lindquist *et al.,* 2016) and peripheral inflammation (Kraynak *et al.,* 2018). The binary maps derived from the meta-analyses were multiplied using *fslmaths* to create a mask that encompassed overlapping voxels from both of the meta-analyses. The regression analyses conducted in this study were restricted to the a priori limited search space represented by the combined meta-analytic mask by specifying this as the pre-threshold mask in FEAT. Thus, regression analyses searched for significant clusters of activity within the search space that were associated with systemic inflammation while controlling for covariates (see below for additional details).

Next, this combined meta-analytic mask was used to guide the selection of ROIs for the functional connectivity analysis. Corticolimbic regions present in the mask included the amygdala, insula, hippocampus, thalamus, striatum, pallidum and mPFC. The amygdala, insula, hippocampus, pallidum and thalamus ROIs for connectivity analyses were derived from the Harvard–Oxford subcortical structural atlas (Desikan *et al.,* 2006). The striatum mask was generated using the Oxford–Imanova striatal structural atlas (Tziortzi *et al.,* 2014). The mPFC mask was generated using the Sallet Dorsal Frontal connectivity–parcellation atlas (Sallet *et al.,* 2013). ROI clusters 3 and 4, which consisted of Brodmann Areas 9 and 10 (Lieberman *et al.,* 2019), were combined to create an mPFC mask.

#### Regression analyses associating levels of inflammation with neural activity

Two general linear models were employed to assess the relationship between inflammation (composite of CRP and IL-6) and activity in clusters encompassed within the combined meta-analytic mask when participants viewed positive (*vs* neutral) and negative (*vs* neutral) images. Consistent with prior work in this area (O’Connor *et al*., 2009a), group-level regression models controlled for age and gender. Considering adipose tissue’s role in systemic inflammation (Mohamed-Ali *et al.,* 1997), body mass index (computed via measures of height and weight) was also included as a covariate. The higher-level models conducted for these analyses utilized cluster-based thresholding at *z* > 2.3, *P* < 0.05. In conjunction with FSL FLAME 1, the correction parameters used in this study have been found to effectively decrease type II errors (Eklund *et al.,* 2016).

#### Functional connectivity analyses

Finally, functional connectivity analyses were conducted utilizing the functional connectivity toolbox (CONN-Toolbox v.18.b; Whitfield-Gabrieli and Nieto-Castanon, 2012). The CONN toolbox was used to perform ROI-to-ROI regression analyses to examine associations between inflammation and corticolimbic ROI connectivity during the two contrasts of interest. Trial onsets and durations were imported into the toolbox to implement the generalized psychophysiological interaction procedure (gPPI; McLaren *et al.*, 2012). Following the standard CONN denoising pipeline, a simultaneous linear regression and temporal band-pass filtering procedure was conducted to remove the influence of non-neural variability in the data (Hallquist *et al.,* 2013). The pipeline implemented an anatomical component-based noise correction process (aCompCor) to remove the first five principal noise components from white matter and cerebrospinal fluid. Twelve motion parameters, outlier scans, constant linear session effects and constant task-related effects were also included as regressors. Finally, temporal frequencies above 0.09 Hz and below 0.008 Hz were removed to minimize further the influence of physiological and motion sources of noise.

While controlling for age, gender and BMI, two separate models examined the association between inflammation and functional connectivity between all possible combinations of the seven ROIs while participants viewed negative (*vs* neutral images) and positive (*vs* neutral images). An analysis-wise false discovery rate (FDR) at *P* < 0.05 was implemented to correct for multiple comparisons.

## Results

### Negative *vs* neutral images

#### Overall neural reactivity

Results from the whole-brain analysis for the negative *vs* neutral contrast identified three significant clusters (*z* > 2.3, *P* < 0.05). Two clusters extended from the lateral occipital cortex, through the middle temporal gyrus, to the inferior temporal gyrus within both hemispheres (*z* = 5.98, *k* = 2794, *P* = 0.0001; *z* = 6.65, *k* = 2615, *P* = 0.0002). The final cluster extended from the left and right amygdala through to the right thalamus (*z* = 4.87, *k* = 2728, *P* = 0.0001). See Supplementary Table S1 for full details.

#### Inflammation and neural reactivity

To examine the relationship between inflammation and neural reactivity to negative images, we ran regression analyses looking for clusters of activity within our search space mask to negative (*vs* neutral) images that were significantly associated with the composite measure of inflammation, controlling for age, gender and BMI. Contrary to hypotheses, we found no significant associations between levels of inflammation and activity in any clusters within the mask when participants viewed negative (*vs* neutral) images.

#### Inflammation and functional connectivity

Next, we conducted an ROI-to-ROI regression analysis to examine the relationship between inflammation and connectivity between the corticolimbic regions of interest in response to negative images. While controlling for age, gender and BMI, inflammation was not significantly associated with connectivity between any of the ROIs (p-FDR > 0.05).

### Positive *vs* neutral images

#### Overall neural reactivity

Results from the whole-brain analysis for the positive *vs* neutral contrast identified three significant clusters. One large cluster encompassed regions in the occipital cortex extending into the putamen, hippocampus and amygdala (*z* = 7.49, *k* = 13 949, *P* < 0.001). Another cluster was found in the vmPFC (*z* = 5.37, *k* = 1726, *P* = 0.0019). A final cluster extended from the cingulate gyrus through to the precuneus (*z* = 4.13, *k* = 1180, *P* = 0.016). See Supplementary Table S2 for full details.

#### Inflammation and neural reactivity

**Fig. 1:**
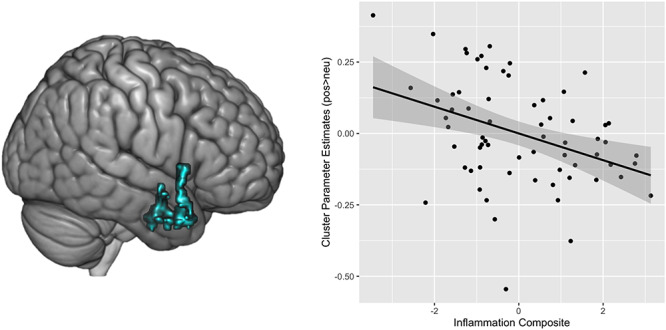
The rendered image on the left depicts the cluster of voxels that showed a significant negative association between inflammation and neural activation to positive (*vs* neutral) images while controlling for age, gender and BMI. The image on the right illustrates a scatterplot of the negative association between composite inflammation and activation in that cluster to the positive (*vs* neutral) images.

Next, we ran regression analyses looking for clusters of activity within our search space mask to positive (*vs* neutral) images that were significantly associated with the composite measure of inflammation, controlling for age, gender and BMI. There was a negative association between inflammation and activation in one cluster, such that higher levels of inflammation were associated with lower levels of activity in a cluster encompassing voxels in the anterior insula, amygdala, hippocampus and temporal pole (peak coordinate: *x* = 30, *y* = −10, *z* = ; *z* = 3.74, *k* = 515, *P* = 0.0134; see Figure 1). See Table 2 for more information regarding the regions encompassing the cluster.

**Table 2:** Local maxima within significant cluster negatively associated with inflammation during positive > neutral images

Region	*x* (mm)	*y* (mm)	*z* (mm)	*Z* statistic
Parahippocampal gyrus	30	−10	−32	3.74
Insular cortex	44	14	0	3.6
Inferior temporal gyrus	44	2	−32	3.51
Temporal pole	34	12	−32	3.29
Right amygdala	26	−3	−23	2.83

#### Inflammation and functional connectivity

**Fig. 2:**
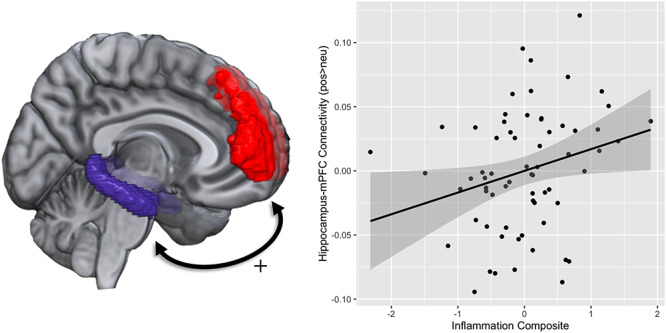
The image on the left depicts the ROI masks of the hippocampus and medial prefrontal cortex that were used for the analyses. The scatterplot on the right illustrates the positive association between inflammation and hippocampus–mPFC connectivity while viewing positive (vs neutral) images.

Next, we conducted an ROI-to-ROI regression analysis to examine the relationship between inflammation and connectivity between the corticolimbic regions of interest in response to positive images. While controlling for age, gender and BMI, the composite inflammation score was positively associated with bilateral hippocampus–mPFC connectivity (*t*(61) = 3.68, p-FDR = 0.028; see Figure 2).

## Discussion

Results from the present study suggest that levels of peripheral inflammation are associated with differences in neural reactivity and connectivity while processing positive affective information among mid- to late-life adults. First, higher levels of peripheral inflammation were associated with lower activation in the amygdala, hippocampus, anterior insula and temporal pole in response to positive images (*vs* neutral). There were no associations between markers of inflammation and neural reactivity to negative images (*vs* neutral). Second, the present study also found that greater inflammation was associated with stronger connectivity between the hippocampus and medial prefrontal cortex in response to positive images (*vs* neutral). Together, these results add to a growing literature in health neuroscience documenting associations between peripheral inflammation and neural responses to social and affective information. The present results extend past findings by looking at an older sample of individuals and novel affective reactivity paradigm while also exploring the associations between inflammation and task-based functional connectivity.

Our first set of findings showing that greater inflammation is associated with lower neural activity in the amygdala, hippocampus, insula and temporal pole activity in response to positive stimuli is consistent with a growing literature documenting associations between inflammation and blunted neural reactivity to positive stimuli (Capuron *et al.,* 2012; Eisenberger *et al*., 2010a; Moieni *et al.,* 2019). Lower reactivity to positive images in canonical regions implicated in the detection of and attention to salient stimuli suggests that less sensitivity to positive stimuli may be linked to higher low-grade inflammation. One possible psychological interpretation of these findings is that inflammation may blunt neural sensitivity to positive experiences, which may generally reduce one’s interest in positive information and motivation to engage with positive stimuli (Eisenberger *et al*., 2017), perhaps in an effort to conserve metabolic resources. Although we do not see inflammation-related differences in activity in the regions implicated in processing reward (e.g. basal ganglia) that other studies have found (Capuron *et al.,* 2012; Eisenberger *et al*., 2010a; Felger and Miller, 2012), our findings are consistent with the general idea that inflammation is related to lower levels of neural activation to positive stimuli. Further, the present findings extend previous literature in this area, which has focused almost exclusively on neural responses to monetary reward tasks (c.f., Inagaki *et al.,* 2015; Muscatell *et al*., 2016b), to document that inflammation is also associated with lower levels of activity in temporal-lobe regions in response to a wider variety of positive stimuli (i.e. pictures of positive scenes). Thus, the present results are consistent with prior research showing that inflammation is associated with reduced neural responsivity to positive stimuli.

Surprisingly, we did not find an association between levels of peripheral inflammation and corticolimbic activation to negative images. This lack of association is inconsistent with prior research, indicating that reactivity to negative stimuli is positively associated with inflammation (e.g. Inagaki *et al.,* 2012; Gianaros *et al.,* 2014; Muscatell *et al.,* 2016a). Though null findings should be interpreted with caution, differences between the current study and past work in this area provide potential explanations for this lack of association. For example, others have found that neural activity to negative stimuli varies as a function of participant age (Mather, 2012), and the present study utilized a mid- to later-later life sample, whereas most other work on associations between inflammation and neural responses to negative stimuli have utilized younger samples (Gianaros *et al.,* 2014; Swartz *et al.,* 2017). Thus, age differences in participants may partially explain the divergence between the present findings and past work in this area. Additionally, others have found that inflammation differentially influences neural activation to social *vs* non-social stimuli (Inagaki *et al.,* 2012) and most of the work in this area examines neural responses to negative social stimuli (e.g. threatening faces, negative social feedback; Inagaki *et al.,* 2012; Muscatell *et al*., 2016a,b; c.f. Gianaros *et al.,* 2014). To conserve power, neural activity to social and non-social images were collapsed in this study, which may also explain the lack of expected associations. Additional work is needed to examine whether there is indeed no association between low-grade inflammation and neural reactivity to negative information broadly or if specific characteristics of the present sample or task contribute to the lack of association observed in the current analysis.

In response to the positive stimuli, inflammation was positively associated with connectivity between the hippocampus and the medial prefrontal cortex. These findings are consistent with past literature, showing that inflammation is associated with differential corticolimbic connectivity (Felger *et al.,* 2016; Kraynak *et al.,* 2018; Kraynak *et al.,* 2019), although the specific pattern of positive associations between inflammation and hippocampal-cortical connectivity conflicts with findings from several resting-state studies. Specifically, among healthy volunteers, markers of inflammation have been shown to be negatively associated with connectivity among corticolimbic regions (Kraynak *et al.,* 2019) and regions in the emotion regulation, central executive and default mode networks (Dev *et al.,* 2017; Marsland *et al*., 2017a; Nusslock *et al.,* 2019). Thus, there may be differential associations between inflammation and corticolimbic connectivity, depending upon if the connectivity is measured at rest or in response to a task.

Not only does the current study suggest the need for studies exploring task-based functional connectivity and inflammation, but it also expands our understanding of the neuro-immune influences on affective reactivity. Results from the current study support the preclinical and clinical studies that implicate the hippocampus as a critical node in neuroinflammatory processes (Colasanti *et al*. 2016; Williamson and Bilbo 2013). Inflammation has been shown to alter hippocampal neurogenesis (Giannakopoulou *et al*., 2013), and synaptic plasticity (Nisticò *et al*., 2013), which may extend to the inflammation-related differences in hippocampal activity and connectivity observed in the current study. Additionally, prior work suggests that hippocampus–mPFC connectivity is critical for cognitive and emotion regulation as well as spatial and emotional memory processes (Jin and Maren, 2015). Further, inflammation has also been implicated in emotion and cognition-related impairment (Appleton *et al.,* 2013; Patki *et al.,* 2013). Though speculative, these results suggest the possibility of a neuro-immune pathway whereby affective and memory-related disruptions relate to inflammatory processes via differences in hippocampal–medial prefrontal connectivity. Although this study provides initial evidence regarding task-based corticolimbic connectivity and inflammation, future studies should explore the links between task-based neural connectivity, inflammation and the behavioral sequelae to expand our understanding of the neuro-immune influences on social and affective processes.

Multiple bidirectional physiological pathways provide plausible mechanisms for the observed links between systemic inflammation and neural activity/connectivity (Irwin and Cole, 2011). First, neural activity can alter peripheral inflammation via ‘top-down’, efferent pathways. Corticolimbic activity in response to negative stimuli can elicit the activation of the sympathetic nervous system and release of catecholamines, which can then lead to greater inflammation (Sternberg, 2006; Nusslock and Miller, 2016). Likewise, more positive affect has been linked with increases in cardiac vagal tone (Kok *et al*., 2013), and the vagus nerve can dampen pro-inflammatory responses (Pavlov and Tracey, 2012; Metz and Tracey, 2005). Second, peripheral inflammation can alter neurotransmitter, neuron and cerebral microvasculature functioning via a ‘bottom-up’, afferent pathway. Cytokines such as IL-6 can reach the central nervous system through active transport, binding to receptors on peripheral nerves (e.g. the vagus nerve), and by crossing the blood–brain barrier in areas of increased permeability (Dantzer *et al.*, 2000; Dantzer *et al*., 2008). As such, systemic inflammation may affect corticolimbic function by entering the central nervous system to alter neurotransmitter (e.g. dopamine) and neuron functioning (Capuron *et al.,* 2012; Felger and Treadway, 2017; Menard *et al.,* 2017). Considering the multiple differing routes by which inflammation and neural activity relate, the precise mechanism linking inflammation and neural responses in this study is unknown. Future work is needed to gain clarity on the specific pathways linking inflammation and neural reactivity to affective information.

The present findings should be interpreted in the context of the study’s limitations. First and foremost, the current study was cross-sectional, which precludes concluding the direction of the association between neural responses and peripheral inflammation. Second, as with many fMRI studies, our project has a relatively small sample size (*N* = 66), and thus future work with larger samples is needed to replicate the findings observed here. Finally, only two markers of systemic inflammation (i.e. CRP and IL-6) were explored in this analysis. Other studies that explore how neural activation varies as a function of a diverse set or pattern of inflammatory markers would be an important contribution to future literature.

In sum, the present project utilized publicly available data from the MIDUS study to bring together methods from psychoneuroimmunology and affective neuroscience to explore a question at the core of health neuroscience research (Erickson *et al.* 2014): How are physiological processes implicated in disease development associated with neural functioning? The results are consistent with theorizing on the neuro-immune network (Nusslock and Miller, 2016), suggesting that inflammation in the periphery is associated with neural activity in and connectivity between regions that are critical for supporting successful social behavior and emotional functioning. More broadly, the present findings highlight the utility of health neuroscience approaches to map the connections between the brain and the body, showing that physiological processes such as inflammation are related to how our brains respond to affective information. As such, physiologic functioning may represent an often-overlooked contributor to and consequence of social and affective processes that social cognitive and affective neuroscience should work to incorporate into future empirical work and theoretical models of functioning within the social brain.

## Supplementary Material

nsaa065_Supp
