# Supplementary material for: Systemic inflammation is associated with differential neural reactivity and connectivity to affective images
Source: Soc Cogn Affect Neurosci. 2020 May 14;15(10):1024–33. doi: 10.1093/scan/nsaa065 (PMC7657451; doi:10.1093/scan/nsaa065)
Supplement: nsaa065_Supp [file nsaa065_supp.zip › scan-19-168-File004_nsaa065.docx]

Supplemental Material for

**Systemic Inflammation is Associated with Differential Neural Reactivity and**

**Connectivity to Affective Images**

Table of Contents

[***Supplemental Methods*** *2*](#_Toc37103846)

[IAPS Image Labels 2](#_Toc37103847)

[Biosamples information 2](#_Toc37103848)

[***Supplemental Results*** *4*](#_Toc37103849)

[Decomposing Inflammation: IL-6 and CRP Specific Analyses 4](#_Toc37103850)

[Voxel-Wise Associations between Inflammation and Affective Reactivity 4](#_Toc37103851)

[***Supplemental Tables*** *5*](#_Toc37103852)

[Table S1: Significant Clusters Activated for Negative > Neutral Contrast 5](#_Toc37103853)

[Table S2: Significant Clusters Activated for Positive > Neutral Contrast 6](#_Toc37103854)

***Supplemental Methods***

**IAPS Image Labels**

Negative: 1111, 1220, 1275, 1525, 2053, 2205, 2278, 2490, 2700, 2717, 2750, 2799, 2800, 3216, 3280, 5973, 6200, 6210, 6250, 6311, 6562, 6570, 6838, 6840, 7359, 7361, 9000, 9001, 9007, 9008, 9090, 9101, 9140, 9182, 9265, 9280, 9290, 9300, 9301, 9320, 9331, 9373, 9419, 9425, 9426, 9471, 9520, 9561, 9570, 9571, 9584, 9600, 9622, 9630, 9810, 9830, 9902, 9903, 9911, 9925

Neutral: 1616, 2038, 2102, 2191, 2200, 2210, 2214, 2215, 2305, 2357, 2385, 2393, 2396, 2397, 2441, 2445, 2480, 2487, 2493, 2499, 2512, 2516, 2579, 2595, 2749, 2840, 2850, 5471, 5520, 5535, 6150, 7006, 7009, 7030, 7036, 7037, 7038, 7041, 7044, 7050, 7100, 7130, 7160, 7161, 7170, 7179, 7180, 7184, 7207, 7235, 7242, 7247, 7249, 7484, 7493, 7500, 7547, 7550, 9070, 9700

Positive: 1463, 1540, 1590, 1650, 1660, 1710, 1721, 1731, 1811, 2030, 2040, 2170, 2216, 2222, 2303, 2306, 2345, 2346, 2391, 4610, 4623, 4625, 4626, 5270, 5450, 5470, 5480, 5600, 5660, 5700, 5820, 5849, 7250, 7280, 7289, 7390, 7400, 7430, 7450, 7470, 7502, 7508, 8040, 8090, 8116, 8120, 8162, 8170, 8200, 8220, 8350, 8370, 8371, 8460, 8461, 8496, 8499, 8502, 8531, 8540

**Biosamples Information**

Samples were stored in a -60 C to -80 C freezer until they were shipped to the MIDUS Biocore Lab (Ryff, Seeman, & Weinstein, 2010). IL-6 assays were conducted using Quantikine High Sensitivity ELISAs (R&D Systems, Minneapolis, MN). The assay sensitivity was 0.16 pg/ml, and the inter-assay coefficient of variation was 12.31%. CRP levels were measured using an immunonepholometric assay. The assay sensitivity was 0.18 ug/ml, and the inter-assay coefficient of variation ranged 2.1-5.7%. Samples that fell below the lower limit of detection using this assay method were then assayed using a high sensitivity immunoelectrochemiluminescence assay (Meso Scale Diagnostics #K151STG). The assay sensitivity was 0.01 ug/ml, and the inter-assay coefficient of variation ranged 4.7-5.2%.

***Supplemental Results***

**Decomposing Inflammation: IL-6 and CRP Specific Analyses**

We examined the separate associations between CRP and IL-6, respectively, and neural reactivity to the positive (vs. neutral) images, to further probe the observed associations between the composite inflammatory score and neutral responses in the limbic cluster. After controlling for age, gender, and BMI, CRP was not significantly associated with activity in the meta-analytic mask. In contrast, IL-6 was negatively associated with activity in a single cluster extending through the hippocampus, amygdala, insula, and temporal pole [peak coordinate: x= 30, y= -10, z= -32; *p*= 0.0134, size(voxels)= 515, z statistic= 3.74].

Next, we examined the separate associations between CRP and IL-6, respectively, and neural connectivity between ROIs when viewing the positive (vs. neutral) images. After controlling for age, gender, and BMI, CRP was not significantly associated with connectivity among the ROIs. In contrast, IL-6 was positively associated with connectivity between the hippocampus and mPFC ROIs [*t*(61)=3.68, p-FDR=0.028].

**Voxel-Wise Associations between Inflammation and Affective Reactivity**

Additionally, we conducted a voxel-wise analysis for the association between composite inflammation and neural activity to negative (versus neutral) and positive (versus neutral) pictures while controlling for age, gender, and BMI. These analyses were thresholded at z > 2.3, p<0.05. We found no significant associations between inflammation and neural activity for either contrast.

***Supplemental Tables***

**Table S1: Significant Clusters Activated for Negative > Neutral Whole-Brain Contrast**

| Region | Size (Voxels) | *p* value | x (mm) | y (mm) | z (mm) | Z statistic |
| --- | --- | --- | --- | --- | --- | --- |
| Cluster 1 | 2794 | 0.0001 | 52 | -62 | -2 | 5.98 |
| Occipital cortex |  |  | 52 | -62 | -2 | 5.98 |
| Middle temporal gyrus |  |  | 60 | -56 | 2 | 5.39 |
| Inferior temporal gyrus |  |  | 46 | -58 | -8 | 5.00 |
| Cluster 2 | 2728 | 0.0001 | -22 | -2 | -18 | 4.87 |
| Left amygdala |  |  | -22 | -2 | -18 | 4.87 |
| Right amygdala |  |  | 22 | -2 | -16 | 4.86 |
| Inferior frontal gyrus |  |  | 52 | 30 | -6 | 4.51 |
| Frontal orbital cortex |  |  | -30 | 18 | -24 | 4.3 |
| Cluster 3 | 2615 | 0.0002 | -54 | -64 | 0 | 6.65 |
| Occipital cortex |  |  | -54 | -64 | 0 | 6.65 |
| Angular gyrus |  |  | -40 | -60 | 18 | 4.26 |

**Table S2: Significant Clusters Activated for Positive > Neutral Whole-Brain Contrast**

| Region | Size (Voxels) | *p* value | x (mm) | y (mm) | z (mm) | Z statistic |
| --- | --- | --- | --- | --- | --- | --- |
| Cluster 1 | 13949 | 3.68e^-16^ | 52 | -70 | 0 | 7.49 |
| Occipital cortex |  |  | 52 | -70 | 0 | 7.49 |
| Middle temporal gyrus |  |  | 56 | -58 | -2 | 5.85 |
| Right Hippocampus |  |  | 33 | -7 | -21 | 3.54 |
| Right Amygdala |  |  | 28 | -3 | -21 | 3.22 |
| Right Putamen |  |  | 30 | -16 | 0 | 3.15 |
| Cluster 2 | 1726 | 0.0019 | -4 | 62 | 2 | 5.37 |
| vmPFC (BA 10) |  |  | -4 | 62 | 2 | 5.37 |
| vmPFC (BA 9) |  |  | 9 | 61 | 14 | 2.86 |
| Cluster 3 | 1180 | 0.016 | -2 | -52 | 20 | 4.13 |
| Cingulate gyrus |  |  | -2 | -52 | 20 | 4.13 |
| Precuneus |  |  | -6 | -58 | 26 | 3.66 |

Reference

Ryff, C. D., Seeman, T., & Weinstein, M. (2010). Midlife in the United States (MIDUS 2): Biomarker Project, 2004-2009. *Inter-University Consortium for Political and Social Research*. https://doi.org/10.3886/icpsr29282.v9
